# Pharmacological Blockade of Serotonin 5-HT_7_ Receptor Reverses Working Memory Deficits in Rats by Normalizing Cortical Glutamate Neurotransmission

**DOI:** 10.1371/journal.pone.0020210

**Published:** 2011-06-20

**Authors:** Pascal Bonaventure, Leah Aluisio, James Shoblock, Jamin D. Boggs, Ian C. Fraser, Brian Lord, Timothy W. Lovenberg, Ruggero Galici

**Affiliations:** Johnson & Johnson Pharmaceutical Research and Development, L.L.C., San Diego, California, United States of America; VU University - Neuroscience Campus, The Netherlands

## Abstract

The role of 5-HT_7_ receptor has been demonstrated in various animal models of mood disorders; however its function in cognition remains largely speculative. This study evaluates the effects of SB-269970, a selective 5-HT_7_ antagonist, in a translational model of working memory deficit and investigates whether it modulates cortical glutamate and/or dopamine neurotransmission in rats. The effect of SB-269970 was evaluated in the delayed non-matching to position task alone or in combination with MK-801, a non-competitive NMDA receptor antagonist, and, in separate experiments, with scopolamine, a non-selective muscarinic antagonist. SB-269970 (10 mg/kg) significantly reversed the deficits induced by MK-801 (0.1 mg/kg) but augmented the deficit induced by scopolamine (0.06 mg/kg). The ability of SB-269970 to modulate MK-801-induced glutamate and dopamine extracellular levels was separately evaluated using biosensor technology and microdialysis in the prefrontal cortex of freely moving rats. SB-269970 normalized MK-801 -induced glutamate but not dopamine extracellular levels in the prefrontal cortex. Rat plasma and brain concentrations of MK-801 were not affected by co-administration of SB-269970, arguing for a pharmacodynamic rather than a pharmacokinetic mechanism. These results indicate that 5-HT_7_ receptor antagonists might reverse cognitive deficits associated with NMDA receptor hypofunction by selectively normalizing glutamatergic neurotransmission.

## Introduction

The 5-HT_7_ receptor, a postsynaptic G protein coupled receptor was identified in 1993 through a homology cloning strategy and found to modulate positive cAMP formation via G_s_ [1]. In the brain, 5-HT_7_ receptors are distributed in the suprachiasmatic nucleus of the hypothalamus, hippocampus, cortex, thalamus and raphe nuclei on GABAergic interneurons or on glutamate terminals [1], [2], [3]. 5-HT_7_ receptor antagonists have been evaluated in several animal models predictive of anxiolytic-and antidepressant-like activity [3], [4], [5]. For example, blockade of 5-HT_7_ receptors in the rat Vogel test increased the number of shocks [6]. In addition, mice treated with 5-HT_7_ receptor antagonists as well as mice lacking the 5-HT_7_ receptors display decreased immobility time in the tail suspension test [7], [8], [9], [10]. These results suggest that blockade of 5-HT_7_ receptors may have anxiolytic and antidepressant activity in humans. 5-HT_7_ receptor antagonists have been also evaluated in animal models predictive of antipsychotic-like activity. One hypothesis of schizophrenia is based on N-methyl-D aspartic acid (NMDA) receptor hypofunction. NMDA antagonists such as ketamine and phencyclidine (PCP) induce hyperactivity, stereotypy and sensorimotor gating deficits in multiple species including humans and they can exacerbate positive symptoms of schizophrenia. Treatments with atypical antipsychotic drugs are known to reverse these effects [11]. Preclinical studies have demonstrated that SB-258741, a selective 5-HT_7_ receptor antagonist, blocked phencyclidine (PCP)-induced hyperactivity in rats [12]. Furthermore, SB-269970, an analog of SB-258741, partially but significantly blocked ketamine -induced hyperactivity in mice [13]. In addition, PCP-induced prepulse inhibition deficits were less pronounced in 5-HT_7_ knockout mice compared to wild-type mice [14]. However, 5-HT_7_ receptor antagonists did not reverse prepulse inhibition deficits in rats and mice [14]. Thus, the antipsychotic-like activity of selective 5-HT_7_ receptor antagonists is weaker than clinically proven antipsychotic drugs[15]. However, current antipsychotics do not show strong efficacy in cognitive deficit associated with psychiatric disorders including depression and schizophrenia.

It is currently debated whether 5-HT_7_ receptor antagonists can improve cognition [16]. Mice lacking the 5-HT_7_ receptor showed impaired contextual fear conditioning but no significant deficits in motor and spatial learning or cued and operant conditioning [17]. Animal studies with selective 5-HT_7_ receptor antagonists have provided mixed results [16]. In one study using the radial arm maze task, 5-HT_7_ receptor blockade had a pro-cognitive effect, when the learning task implicated a high degree of difficulty [18]. Another study showing that the 5-HT_7_ antagonist SB-269970 did not have any effect on an associative learning task in normal animals [19]. However, in the later study, SB-269970 reversed the amnesic effect elicited by scopolamine (a non selective muscarinic antagonist), MK-801 (dizocilpine, a non-competitive NMDA receptor antagonist) andmCPP (m-chlorophenylpiperazine, a non selective serotonin ligand) [19].

Altered acetylcholine neurotransmission induced by muscarinic antagonist (e.g. scopolamine) and hypofunction of NMDA receptors induced by NMDA antagonists (e.g. MK-801) are associated with cognitive-related deficits in many species including humans [20], [21]. For example, MK-801 and ketamine increase errors in the 8-arm maze delayed-matching to position and autoshaping learning task in rodents [19], [22]. Similarly, in humans, NMDA receptor antagonists impair performance in the Wisconsin Card Sort task as well as other cognitive models [21], [23], [24], [25]. NMDA antagonists increase both glutamate and dopamine extracellular levels in the rat cortex, a brain region critical in mediating cognitive-related responses, particularly working memory and executive function which are impaired in schizophrenic patients [26], [27]. It is thought that NMDA antagonists produce schizophrenic-like reactions in humans by disrupting cortical networks and enhancing glutamate release. Antipsychotics attenuate MK-801-induced glutamate release in the cortex [28], which may mediate some aspects of their clinical efficacy. It is also known that working memory performance is highly dependent on an optimal amount of dopamine in the prefrontal cortex, which tightly regulates network activity [29]. Thus, too little or too much dopamine interferes with working memory performance. MK-801, which enhances dopamine release in the cortex, would thus be expected to interfere with working memory, in part through an over-stimulation of the cortical dopamine system and subsequent disruption to organized network activity.

The goal of the present studies was two-fold: first, to evaluate the effects of the selective 5-HT_7_ receptor antagonist SB-269970 [30], in a translational behavioral model of working memory. The second goal was to evaluate, at a neurochemical level, the effects of SB-269970 on cortical glutamate and dopamine extracellular levels. Specifically, the effect of SB-269970 was evaluated in rats using delayed non-matching to position (DNMTP), a translational procedure that is thought to measure working memory. In addition, the effects of SB-269970 on glutamate and dopamine extracellular levels were evaluated using biosensor technology (glutamate) and microdialysis (dopamine) in freely moving rats. A pharmacokinetic study was also carried out to determine plasma and brain concentrations of MK-801 after co-administration of SB-269970 to investigate a potential drug-drug interaction.

## Methods

### Ethics Statements

All animal experiments were carried out in accordance with the National Institutes of Health Guide for the Care and Use of Laboratory Animals (NIH Publications No. 8023, revised 1978) and were approved by the Institutional Animal Care and Use Committee (IACUC) at Johnson & Johnson Pharmaceutical Research & Development, L.L.C., San Diego (microdialysis protocol #1048, behavior protocol #100063, pharmacokinetics protocol # 1000117).

### Drugs

SB-269970 hydrochloride ((2R)-1-[(3-hydroxyphenyl)sulfonyl]-2-[2-(4-methyl-1-piperidinyl)ethyl]-pyrrolidine) was purchased from Tocris Bioscience (Ellisville, MO). MK-801 ((5S,10R)-(+)-5-Methyl-10,11-dihydro-5H-dibenzo[a,d]cyclohepten-5,10-imine maleate) and scopolamine were purchased from Sigma Aldrich (St. Louis, MO). All compounds were dissolved in saline (SB-269970, MK-801) or water (scopolamine). Doses are expressed as mg/kg of free base (volume of injection  = 1 ml/kg).

### Delayed non-matching to position (DNMTP) task

Sprague-Dawley rats (Harlan, Indianapolis, Ind., USA, 250–300 g upon arrival) were maintained at 80–90% of their free feeding weight. All animals were single-housed in plastic cages under a 12h:12hlight:dark schedule in humidity controlled rooms and had ad libitum access to water. Animals were trained to respond for food in sound attenuating operant chambers (Med-Associates, St Albans, VT). Chambers were equipped with two levers with stimulus lights and a food hopper. Each test session lasted 30 min and started with the illumination of the house-light and the random presentation of one lever. When the animal pressed the lever, it was immediately retracted for up to a random period of time (0, 4, 8, 16, or 32 sec delay). At the end of the delay both levers were presented. Pressing the lever which was not initially presented (i.e. correct response) resulted in the illumination of both stimulus lights for 1 s, the delivery a 45 mg food pellet (Bio-Serv, Frenchtown, NJ, USA), and the end of the trial. Pressing the lever that was initially presented (incorrect response) resulted in the retraction of both levers and the end of the trial. At the end of the trial, a new trial with a new random delay was initiated. SB-269970 (10 mg/kg) and scopolamine (0.06 mg/kg), or their vehicles, were administered intraperitoneally at 10 and 30 min, respectively, before the beginning of the session. SB-269970 (10 mg/kg) and MK-801 (0.1 mg/kg), or their vehicles, were co-administered intraperitoneally at 10 min before the beginning of the session. The dose of MK-801 was chosen based on pilot studies showing impaired working memory without producing gross psychostimulant effects that would interfere with lever pressing. Each experiment was conducted in a Latin-square design with washout periods between each drug test (N = 6). Data from each experiment were analyzed for percent correct choices with a two-way analysis of variance (ANOVA) for treatment group x delay, with repeated measures on delay, followed by Duncan's post-hoc tests when appropriate. The number of trials completed during each session was analyzed using a one-way repeated measures ANOVA for treatment group. Statistica (StatSoft Inc., Tulsa OK) was used for data analysis.

### Real-time measurements of glutamate efflux in the cortex of freely moving rats

Male Sprague-Dawley rats (Charles River Laboratories) weighing 280–350 grams were used. All animals were single-housed in a 12h:12hlight:dark schedule in humidity controlled rooms and had ad libitum access to water. Each rat was given a subcutaneous 0.05 mL injection of Buprenex (buprenorphine hydrochloride; Reckitt Benckiser Pharmaceuticals Inc., Richmond, VA) at 0.06 mg/kg 5 minutes prior to anesthesia. Animals were anesthetized with an Isoflurane/air mixture and stereotaxically implanted with a guide cannula (BAS) in the prefrontal cortex (incisor bar, −3.5 mm, 3.2 mm anterior, 0.9 mm lateral and 2 mm ventral to Bregma, [31]. The guide cannula and bone screws were encased with acrylic dental cement. Animals were allowed at least 5 days to recover from surgery prior to experimentation. The animals were handled each day and experimentation occurred within the animal's home cage.

The glutamate biosensor (wireless model 7001, Pinnacle Technologies, Lawrence KS) specification and hardware setup was described previously [32]. Prior to sensor insertion, each biosensor was calibrated in-vitro to verify glutamate sensitivity and interference rejection. The morning of experimentation, under light isoflurane anesthesia, the biosensor was inserted into the prefrontal cortex. Experimentation began once a stable sensor signal was obtained, approximately 4 hours. At the completion of the experiment, each sensor was removed from the guide cannulae and calibrated *in vitro* for glutamate sensitivity and interference rejection of ascorbate at 37°C in a circulating water bath.

Initially, all 15 animals received an intraperitoneal vehicle injection. Sixty min later, they received the following intraperitoneal treatments: vehicle + MK-801, 0.1 mg/kg (N = 5), SB-269970, 10 mg/kg + MK-801, 0.1 mg/kg (N = 5) or SB-269970, 10 mg/kg + vehicle (N = 5).

Following experimentation the data was exported to excel and binned into 5 minute current (nA) intervals. These data points were then converted to glutamate concentrations using calibration curve regression data for the corresponding sensor.

Statistical analyses were performed on the change in glutamate concentrations with a two-way ANOVA for treatment x time, with repeated measures on time, followed by Duncan's post-hoc tests. Statistics were calculated using Statistica (StatSoft Inc., Tulsa OK).

### Dopamine microdialysis in the cortex of freely moving rats

Male Sprague-Dawley rats (Charles River Laboratories) weighing 280–350 grams were used. All animals were single-housed in a 12h:12hlight:dark schedule in humidity controlled rooms and had ad libitum access to water. Each rat was given a 0.05 ml SC injection of Buprenex 0.06 mg/kg (buprenorphine hydrochloride) 5 mins prior to anesthesia. Animals were anesthetized with an isoflurane/air mixture and stereotaxically implanted with a guide cannulae (Eicom) in the prefrontal cortex (incisor bar −3.5 mm, +3.2 mm anterior, 0.8 mm lateral and 1 mm ventral to Bregma) [31]. The guide cannula was secured in place with skull screws and dental cement. Animals were allowed at least 3 days to recover from surgery prior to experimentation.

Dialysis experiments were conducted as previously described [33]. Dialysate was analyzed by high-performance liquid chromatography with an electrochemical detector (Eicom HPLC/EC). The dopamine concentration for each sample was calculated from the peak area of the chromatographic signal and the slope from the corresponding standard curve.

A between subject design was used. The following conditions were evaluated: vehicle + vehicle (N = 5), vehicle + MK-801, 0.1 mg/kg (N = 6), SB-269970, 10 mg/kg + MK-801, 0.1 mg/kg (N = 5) and SB-269970, 10 mg/kg + vehicle (N = 5). All compounds were administered intraperitoneally.

The percent change from baseline values were calculated from the mean basal value of each neurotransmitter for each animal and presented in the figures as mean ± S.E.M.

Statistical analyses were performed on the percent baseline with a two-way ANOVA for treatment x time, with repeated measures on time, followed by Duncan's post-hoc tests. Statistics were calculated using Statistica (StatSoft Inc., Tulsa OK).

At the end of the experiments, microdialysis and biosensor probe placement was visually verified for each animal. Brains were removed, frozen and sectioned with a microtome to verify probe placement. Only animals with correct placement of the probe were used for data analysis.

### Pharmacokinetics and bioanalysis

Male Sprague-Dawley rats (Charles River Laboratories) weighing 300–350 grams were used. Intraperitoneal dosing (vehicle +0.1 mg/kg MK-801 or 10 mg/kg SB-269970 +0.1 mg/kg MK-801; N = 4 per group) was followed by blood sampling via cardiac puncture over a time course. Brains were removed from the animals and homogenized for LC/MS-MS analysis. All blood samples were deproteinized by 1∶4 dilution of the sample with acetonitrile with vigorous mixing. These samples were incubated for 5 minutes, and then centrifuged at 14,000 rpm in a micro-centrifuge for 4 minutes. The supernatant was recovered into auto-sampler vials and diluted 1∶1 with sterile water. Samples were analyzed by LC-MS/MS. A Vydac SP C18 2.1×50 mm analytical column was used for separation.

Statistics (paired t-test) were calculated using Prism software (GraphPad, San Diego, CA). The level of significance was p<0.05. A one compartmental pharmacokinetic model was applied to these data using the software package WinNonlin Version 4.0.1. (Pharsight, Palo Alto, Ca). A one compartment first order, no lag time, first order elimination model (Model 3) was employed. The parameters of the model were optimized using least squares non-linear regression. Pharmacokinetic and blood brain barrier penetration parameters are given as the means ± coefficient of variation. The coefficient of variation is a measure of dispersion of a probability distribution. It is defined as the ratio of the standard deviation to the mean. The coefficient of variation was calculated as the ratio of the standard error for each parameter to its estimated value.

## Results

### SB-269970 blocks MK-801 induced deficits in the DNMTP task

**Figure 1:**
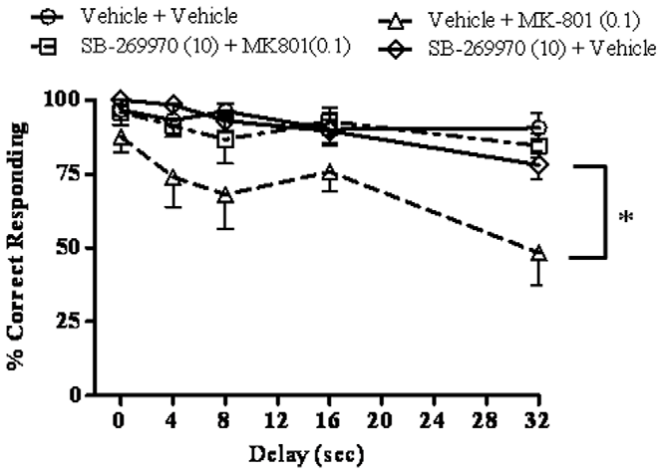
Effects of SB-269970 on MK-801-induced DNMTP deficits. SB-269970 (10 mg/kg) and MK-801 (0.1 mg/kg), or their vehicles, were co-administered 10 min before the beginning of the session (N = 6 per group). SB-269970 had no effect on its own but reversed the deficit caused by MK-801 (*p<0.05, main effect). For statistical details see the result section.

The effect of SB-269970 (10 mg/kg, IP) on MK-801 (0.1 mg/kg, IP) induced deficits was evaluated in the DNMTP task (Fig. 1). The number of trials completed for each group is given in Table 1. A two-way repeated measures ANOVA (delay x treatment group), with repeated measures on group, detected a main effect of delay (F(4, 25) = 5.99, p = 0.0016) and group (F(3, 75) = 15.49, p = 0.000005), but no interaction (F(12, 75) = 1.02, p = 0.44). Duncan's post-hoc tests on group revealed that MK-801 worsened performance (p<0.00005 comparing vehicle + vehicle to vehicle + MK-801). SB-269970 had no effect on its own (p = 0.68, comparing vehicle + vehicle to SB-269970 + vehicle), but reversed the deficit caused by MK-801 (p<0.0002 comparing vehicle + MK-801 to SB-269970 + MK-801) so that the SB-269970 + MK-801 group was no different from the SB-269970 + vehicle group (p = 0.70). There was no difference in the number of trials completed in each session among treatment groups (F(3,15) = 2.42, p = 0.11).

**Table 1:** Number of trials completed in the DNMTP task.

MK-801 (0.1 mg/kg)					Scopolamine (0.06 mg/kg)			
	Vehicle + Vehicle	Vehicle + MK-801	SB-269970 + MK-801	SB-269970 + Vehicle	Vehicle + Vehicle	Vehicle + Scopolamine	SB-269970 + Scopolamine	SB-269970 + Vehicle
Number of trials	48±6	46±8	53±5	40±5	44±5	43±4	35±8	53±5

SB-269970 (10 mg/kg) and MK-801 (0.1 mg/kg), or their vehicles, were co-administered 10 min before the beginning of the session. SB-269970 (10 mg/kg) and scopolamine (0.06 mg/kg), or their vehicles, were administered 10 and 30 min, respectively, before the beginning of the session. There was no difference in the number of trials completed in each session among treatment groups. Data are expressed as mean ± S.E.M., N  =  6 per group).

### SB-269970 does not block scopolamine-induced deficits in the DNMTP task

**Figure 2:**
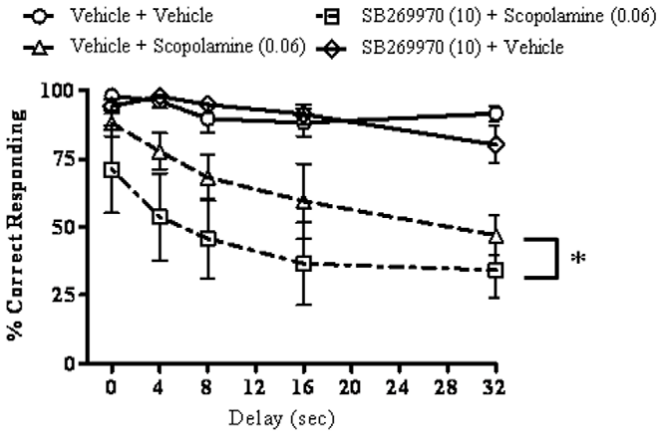
Effects of SB-269970 on scopolamine-induced DNMTP deficits. SB-269970 (10 mg/kg) and scopolamine (0.06 mg/kg), or their vehicles, were administered 10 and 30 min, respectively, before the beginning of the session (N = 6 per group). SB-269970 augmented the deficit caused by scopolamine (*p<0.05, main effect). For statistical details see the result section.

The effect of SB-269970 (10 mg/kg, IP) on scopolamine (0.06 mg/kg, IP) -induced deficits was evaluated in the DNMTP task (Fig. 2). A two-way repeated measures ANOVA (delay x treatment group), with repeated measures on delay, detected a main effect of delay (F(4, 25) = 2.79, p = 0.048) and group (F(3, 75) = 37.29, p = 0.000005), but no interaction (F(12, 75) = 0.88, p = 0.57). Duncan's post-hoc tests on group revealed that scopolamine worsened performance in both the vehicle-pretreated and SB-269970- pretreated groups (p<0.00006 comparing vehicle + vehicle to vehicle + scopolamine and p<0.00005 comparing vehicle + vehicle to SB-269970 + scopolamine). SB-269970 had no effect on its own (p = 0.83, comparing vehicle + vehicle to SB-269970 + vehicle), but augmented the deficit caused by scopolamine (p<0.0003 comparing vehicle + scopolamine to SB-269970 + scopolamine). There was no difference in the number of trials completed in each session among treatment groups (F(3,15) = 2.65, p = 0.086).

### SB-269970 blocks MK-801-induced glutamate efflux in the cortex of freely moving rats

**Figure 3:**
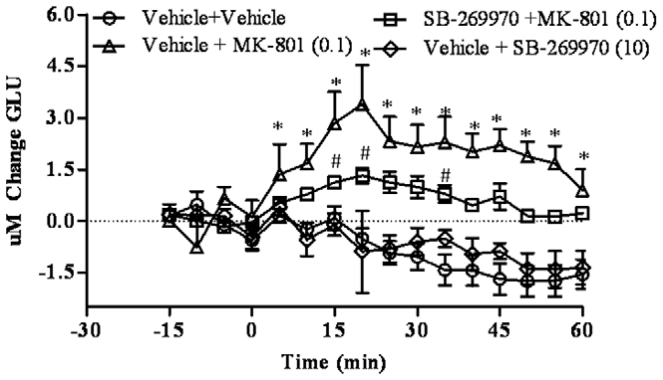
Effects of SB-269970 on MK-801-induced glutamate efflux in the prefrontal cortex. Real-time measurements of glutamate were conducted before and after compound administration. Results are expressed as as mean ± S.E.M of change in glutamate concentrations (N = 5–15 per group). SB-269970(10 mg/kg) did not affect glutamate levels per se but attenuated MK-801(0.1 mg/kg)-induced increases in glutamate efflux. *p<0.05, at each time point comparing vehicle + MK-801 to vehicle + vehicle. ^#^ p<0.05, at each time point comparing SB-269970 + MK-801 to vehicle + MK-801.

The effect of SB-269970 (10 mg/kg, IP) on MK-801 (0.1 mg/kg, IP) induced glutamate efflux was evaluated in the cortex of conscious rats using biosensor technology (Fig. 3.). A two-way repeated measures ANOVA (group x time) revealed a main effect of group (F(3, 26) = 12.45, p = 0.00003) and time (F(15, 390) = 4.31, p = 0.000005), and an interaction (F(45, 390) = 4.34, p = 0.000005). Post-hoc tests revealed that the vehicle + MK-801 had higher glutamate concentrations compared to the vehicle + vehicle group from 10–60 min (p = 0.000001–0.04 at those time points). SB-269970 did not affect glutamate levels per se (p>0.05 at all time points), but attenuated MK-801-induced increases in glutamate (p<0.05 at 15 min, p<0.02 at 20 min, p<0.04 at 35 min, comparing SB-269970 + MK-801 to vehicle+MK-801). Glutamate levels were only elevated in the SB-269970 + MK-801 group compared to the SB-269970 + vehicle group at 20 and 25 min (p<0.02 and p<0.04, respectively).

### SB-269970 does not block MK-801-induced dopamine extracellular levels in the cortex of freely moving rats

**Figure 4:**
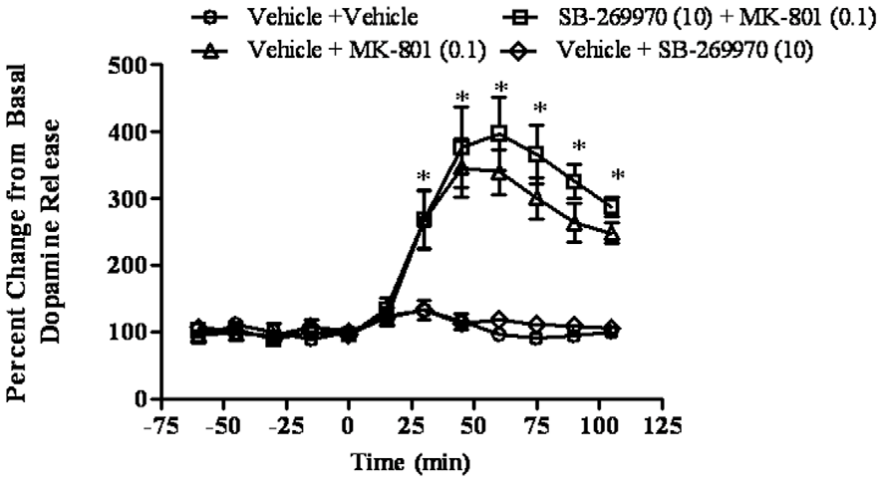
Effects of SB-269970 on MK-801-induced dopamine release. Results are expressed as percentage ofbaseline (mean ± S.E.M., N = 5-6 per group). SB-269970 (10 mg/kg) did not affect dopamine levels by itself, nor did it alter the effect of MK-801 (0.1 mg/kg) on dopamine levels.*p<0.001, at each time point compared to control.

The effect of SB-269970 (10 mg/kg, IP) on MK-801 (0.1 mg/kg, IP) induced dopamine extracellular levels was evaluated in the cortex of conscious rats using microdialysis (Fig. 4.). A two-way repeated measures ANOVA revealed a main effect of group (F(3, 17) = 21.18, p = 0.00001) and time (F(11, 187) = 45.27, p = 0.00005), and an interaction (F(33, 187) = 13.89, p = 0.00005). Post-hoc tests revealed that the vehicle + MK-801 had higher dopamine levels compared to the vehicle + vehicle group from 30 – 105 min (p = 0.000004–0.000075 at those time points). SB-269970 did not affect dopamine levels by itself, nor did it alter the effect of MK-801 on dopamine levels (p>0.05 at all time points).

### SB-269970 does not change MK-801 plasma and brain concentrations

MK-801 plasma and brain concentrations were determined following intraperitoneal dosing of vehicle + MK-801 (0.1 mg/kg) or co-administration of MK-801 (0.1 mg/kg) with SB-269970 (10 mg/kg). The concentration data was analyzed using a one compartment model with WinNonlin to determine pharmacokinetic parameters (Table 2). A t-test was performed on the pharmacokinetic parameters showed that SB-269970 did not affect the kinetics of MK-801 (p>0.05 Table 2).

**Table 2:** Pharmacokinetic and blood brain barrier parameters of MK-801 (0.1 mg/kg, IP) co-administered with vehicle or with SB-269970 (10 mg/kg, IP).

	Vehicle + MK-801	SB-269970 + MK-801
**Plasma**		
T_max_ (h)	0.31±32%	0.31±35%
C_max_ (µM)	0.04±14%	0.03±19%
AUC_inf_ (h µmol/L)	0.08±33%	0.04±25%
**Brain**		
T_max_ (h)	0.40±20%	0.37±28%
C_max_ (µM)	0.28±11%	0.20±15%
AUC_inf_ (h µmol/L)	0.60±23%	0.33±21%

Data are expressed as mean ± coefficient variation (%) (N  = 4 per group).

## Discussion

The results of the present work indicated that the selective 5-HT_7_ receptor antagonist SB-269970 reversed MK-801 but not scopolamine -induced cognitive deficit in the DNMTP task. In addition, the 5-HT_7_ receptor antagonist normalized MK-801-induced glutamate efflux but not MK-801-induced dopamine extracellular levels in the cortex of freely moving rats. The pharmacokinetic study showed that rat plasma and brain concentrations of MK-801 were not affected by co-administration of SB-269970.

The 5-HT_7_ receptor is expressed in brain regions involved in learning and memory such as hippocampal formation and frontal cortex [3]. Several reports in the literature suggest that it may play a role in the control of learning and memory processes [16], [34], [35], [36]. In this study we evaluated the effect of a selective 5-HT_7_ receptor antagonist in the DNMTP task, a translational assay of working memory. As previously reported, MK-801 and scopolamine were found to decrease percent correct responding without affecting the number of trials [20], [37], [38], [39], [40]. The selective 5-HT_7_ receptor antagonist, SB-269970, had no effect by itself on percentage correct responding and number of trials. However, SB-269970 significantly reversed MK-801 but not scopolamine-induced memory deficits. The dose and pretreatment time of SB-269970 selected for this study were based on previous studies where it was demonstrated that the compound is biologically active in other behavioral assays [3], [7], [13], [30]. For example, SB-269970 at a dose of 10 mg/kg was found to reduce immobility time in the tail suspension test and decrease REM sleep duration [7]. In the present study we carried out a pharmacokinetic study to show that rat plasma and brain concentrations of MK-801 were not affected by co-administration of SB-269970, demonstrating that the effect observed in the DNMTP task is not confounded by a drug-drug interaction.

The reversal of MK-801 induced deficits by the 5-HT_7_ receptor antagonist in the DNMTP task observed in the present study is consistent with the study of Meneses showing that SB-269970 reversed MK-801-induced amnesia in the autoshapingPavlovian/instrumental learning task [19]. In the later study, SB-269970 was also found to reverse scopolamine -induced deficit in the same procedure. In our study, using a different experimental paradigm, SB-269970 was found to augment scopolamine -induced deficits in the DNMTP task. It should be noted that the experimental conditions were different between our study and Meneses's study and could have contributed to the contrasting findings. For example, the dose of scopolamine was different (0.17 vs 0.06 mg/kg in our study) and a different strain of rat was used (Wistar in Meneses's study vs Sprague-Dawley in our study).In addition, different forms of memory were being measured (consolidation memory in the Meneses's study, with drug effects measured 24 hours later, and working memory in the present study, with drug effects measured during the test while the drugs were on board). Interestingly, in the present study, SB-269970 shifted the scolopamine delay-response curve straight down, even at the longer delay, where the scolopamine animals were already performing at 50%. Fifty percent represents chance performance, but the SB-269970+scolopamine treated animals were performing at 36.5 and 34.1% at the two longest delays. Performance below chance suggests the animals were specifically choosing the same lever as presented at the start of thetrial, instead of the opposite lever or choosing randomly. Such performance could represent an inability to disengage attention from the first presented lever and might represent an over-compensation for the attention-impairing effects of scolopamine. Such effects would not be observed in the Meneses's study, where consolidation memory was measured and neither drug was on board during the subsequent test. Further neurochemical investigations on the effect of SB-269970 on the level of acetylcholine are needed to shed light on this finding. Potential drug-drug interaction between SB-269970 and scopolamine should be investigated as well. On the other hand, it is also possible that the 5-HT_7_ receptor has more of a modulatory function, with positive or negative effects on memory performance depending on the background state. Noteworthy, 5-HT_7_ receptor antagonists were found to decrease the memory enhancing effects of the 5-HT_1A/7_ agonist 8-OH-DPAT in the autoshapingPavlovian/instrumental learning task to levels far below that of controls, such that performance was even worse than in the vehicle-treated controls [19]. While the 5-HT_7_ receptor antagonists had no effect by themselves per se, they were able to worsen memory performance, but only under the background of 5-HT_1A/7_ activation (which by itself improved performance). Interestingly, in the same study the 5-HT_7_ receptor antagonists were also able to reverse amnesic-inducing effects while being inactive alone [19]. It was hypothesized that under procognitive or amnesic conditions 5-HT_7_ receptor activity would be unmasked, leading to a modulatory role in memory formation [19]. In fact, the mRNA expression of the 5-HT_7_ receptor has been shown to be enhanced during memory consolidation [36], suggesting that 5-HT_7_ receptor activity can be important to normal memory functions. In this study it was also shown that an agonist of the 5-HT_7_ receptor (AS-19) improved memory consolidation, while partially downregulating 5-HT_7_ mRNA expression, whereas amnesic-drugs strongly downregulated 5-HT_7_ mRNA expression. AS-19 reversed the effects of the amnesic-drugs on both memory performance and mRNA expression. Thus, it is possible that a nominal amount of 5-HT_7_ receptor activity is required for normal memory processes and that 5-HT_7_ receptor ligands can have procognitive or amnesic effects (depending on the background level of 5-HT/5-HT_7_ activity) once 5-HT_7_ receptors are unmasked.

A recent study by Horiguchi and Meltzer [41] showed that selective 5-HT_7_ antagonism had a procognitive effect on the PCP-induced impairment in novel object recognition test, a rodent model of declarative memory deficit in schizophrenia. In addition, preferential 5-HT_7_ antagonism was also found to contribute to the ability of the atypical antipsychotics drugs lurasidone and amisulpride to ameliorate the PCP-induced novel object recognition deficit. Another recent study by Horisawa and colleagues also showed that the selective 5-HT_7_ receptor antagonist (SB-656104) and lurasidone reversed MK-801-induced learning impairment in the Morris Water Maze test [42]. Consistent with these finding, Gasbarri and colleagues demonstrated that 5-HT_7_ receptor blockade enhanced memory retention in the radial maze task [18]. In contrast to their previous results [19], Perez-Garcia and Meneses suggested that 5-HT_7_agonism may promote memory in the hypoglutamatergic rat brain [34]. However, information about the selectivity and brain penetration of the 5-HT_7_ agonist (AS-19) used in the later study is lacking, hampering the full interpretation of these data [34]. Another study by Ballaz and colleagues showed that a high dose of SB-269970 caused impairment in novel object recognition in low but not high responder animals suggesting that 5-HT_7_ blockade removed phenotypic differences in novel object discrimination performance [43]. The authors concluded that these findings might indicate different levels of 5-HT_7_ receptor activity, suggesting that the manner in which rats adapt to the environment may be under the influence of the 5-HT_7_ receptor.

In the present study, biosensor and microdialysis studies in the cortex of freely moving rats were conducted to further investigate the neurochemical interaction between 5-HT_7_ and NMDA receptors. The biosensor technique is very comparable to the microdialysis technique and both are able to selectively monitor glutamate levels in the extracellular space. Compared to microdialysis, biosensors are slightly smaller, and may result in less damage around the recording area, and offer a much greater time resolution. Theoretically, both microdialysis and biosensors decrease the concentration of glutamate around the recording area – microdialysis by sampling and removing glutamate from the extracellular fluid and biosensors by converting glutamate to alpha-ketoglutarate. Another difference between the biosensor methods and conventional microdialysis methods is that in microdialysis, the animal is tethered so that tubing can deliver the CSF. In biosensor studies, the animals are completely free moving and untethered, and the biosensor signal is transmitted wirelessly. This may result in less stress to the animal during testing. Using the biosensor technology, MK-801 was found to increase glutamate efflux in the cortex in agreement with the literature [32], [44]. By itself SB-269970 did not modify glutamate or dopamine extracellular levels in cortex. SB-269970 was found to significantly reduce MK-801-induced glutamate efflux. These effect were qualitatively similar to the results obtained with the mGluR2/3 receptor agonist LY-379268 [44].

In contrast, SB-269970 did not modify MK-801 induced dopamine extracellular levels in cortex indicating that blockade of 5-HT_7_ receptors selectively reduced cortical glutamate outflow.

Collectively, the results from the present study indicated that a selective 5-HT_7_ receptor antagonist might reverse cognitive deficits, particularly those associated with NMDA receptor hypofunction, by selectively normalizing glutamatergic neurotransmission. Selective 5-HT_7_ antagonism may represent a novel approach for improvement of cognitive impairments in schizophrenia, particularly working memory.
